# Inhomogeneity of lung ventilation in children with obesity and its potential role in worsening asthma

**DOI:** 10.14814/phy2.70257

**Published:** 2025-04-17

**Authors:** Ahmed Abushahin, Mutasim Abu‐Hasan, Harshita Shailesh, Belavendra Antonisamy, Yahya Hani, Abidan Muhayimana, Majed Al Theyab, Ibrahim Janahi

**Affiliations:** ^1^ Department of Pediatric Medicine, Division of Pulmonology Sidra Medicine Doha Qatar; ^2^ Clinical Pediatrics Weill Cornel Medicine‐Qatar (WCM‐Q) Doha Qatar; ^3^ College of Medicine, Qatar University Doha Qatar

**Keywords:** asthma, children, lung clearance index, lung function, obesity, ventilation inhomogeneity

## Abstract

Asthma is more frequent and severe in individuals with obesity compared to those with normal weight. While inhomogeneity of lung ventilation due to distal airway obstruction is a key feature in asthma, the effect of obesity on ventilation homogeneity is unclear. We conducted a cross‐sectional study comparing lung clearance index (LCI) using multiple breath nitrogen washout technique between children with normal weight and asthma (*n* = 97), overweight/obesity and asthma (*n* = 100), overweight/obesity and no asthma (*n* = 100), and children with normal weight and no asthma (*n* = 67). Spirometry, lung volumes, and fractional exhaled nitric oxide (FeNO) were obtained for comparison. Results showed no significant difference in LCI between overweight/obesity groups and normal weight groups and no significant correlation between LCI and body mass index (BMI). However, LCI was higher in the asthma groups compared to non‐asthma groups (*p* = 0.017, *p* = 0.003). There was a significant negative correlation between LCI and FEV1% predicted, FEV1/FVC, and FEF25–75% predicted (*r* = −0.24, *p* < 0.001; *r* = −0.26, *p* < 0.001; *r* = −0.23, *p* < 0.001), and a positive correlation with RV/TLC (r = 0.17, *p* = 0.003) and FeNO (r = 0.29, p < 0.001). These findings indicate that obesity does not affect the homogeneity of lung ventilation. Therefore, alternative mechanisms should be considered to explain the association between asthma and obesity.

## 1 INTRODUCTION

Asthma and obesity are common health problems in children and adults, with increased prevalence worldwide (Reyes‐Angel et al., 2022; Sutherland, 2014). There is a strong association between obesity and asthma, but the mechanisms driving this association are not fully understood (Di Cicco et al., 2023; Peters et al., 2018). Obesity can affect asthma pathophysiology through different mechanisms, including alteration of respiratory system mechanics, metabolic dysregulation, systemic inflammation, gastroesophageal reflux, and gut microbiome dysbiosis (Reyes Noriega et al., 2023; Reyes‐Angel et al., 2022).

Obesity can affect chest wall as well as airway mechanics. Obesity decreases chest wall compliance, causing reduced lung volumes (Hegewald, 2021). Adults with obesity have reduced forced vital capacity (FVC) and reduced forced expiratory volume in 1 s (FEV1) but normal FEV1/FVC, indicating a restrictive pattern. This decrease in FVC and FEV1 correlates negatively with body mass index (BMI) (Dixon & Peters, 2018; Mafort et al., 2016). Obesity can also cause a reduction in resting lung volumes, including functional residual capacity (FRC), expiratory reserve volume (ERV), and residual volume (RV), consequently leading to reduced airway caliber or even complete closure of small airways (Dixon & Peters, 2018; Shah & Kaltsakas, 2023). Studies using impulse oscillometry showed a positive correlation between BMI and increased small airway resistance at 5 Hz (Pellegrino et al., 2014). The reduced airway caliber can potentially cause inhomogeneity in lung ventilation similar to that seen in individuals with asthma (Arismendi et al., 2020; Melo et al., 2014; Pellegrino et al., 2014).

Obesity in children, on the other hand, is shown to be associated with normal or high FVC and FEV1, but a low FEV1/FVC ratio. (Arismendi et al., 2020) This unique pattern has been attributed to airway dysanapsis, a term coined by Green et al., which describes an imbalance between the growth of lung parenchyma relative to airway caliber, resulting in normal/high FVC and normal/high FEV1 but a low FEV1/FVC ratio (Arismendi et al., 2020; Forno et al., 2017). Some studies link this airway dysanapsis in children with obesity and asthma to airway hyperreactivity, airway inflammation, and a poor response to inhaled corticosteroids (Arismendi et al., 2020; Forno et al., 2017; Jung et al., 2022). The potential effect of airway dysanapsis on the inhomogeneity of lung ventilation is unknown.

Asthma is associated with airway inflammation, airway hyperresponsiveness, and airway obstruction, leading to inhomogeneity in lung ventilation (Levy et al., 2023). Biopsy samples obtained from children with asthma have shown extensive structural changes in the small airways (Dolhnikoff et al., 2009; Nuttall et al., 2021). Spirometry is routinely used to detect airway obstruction parameters such as FEV1, FEV1/FVC, and FEF 25–75% (Levy et al., 2023). However, the correlation between these spirometric parameters of airway obstruction, asthma symptoms, and markers of airway inflammation is reportedly poor (Bacharier et al., 2004; Strunk et al., 2003). Furthermore, FEV1 detects both large and small airway obstruction but is insensitive in detecting mild airway dysfunction and the resulting inhomogeneity of lung ventilation (Horsley, 2009; Keen et al., 2011; Lu et al., 2018). The lung clearance index (LCI) is a more direct measure of the inhomogeneity of lung ventilation due to airway obstruction (Nuttall et al., 2021). LCI is a global index of gas mixing that is obtained using the multiple breath washout (MBW) technique by measuring the lung's efficacy in clearing either a known concertation of an inhaled inert gas such as sulfur hexafluoride (SF6), or the lung's nitrogen during tidal breathing. Any inhomogeneity in lung ventilation will decrease the lung's efficacy in clearing these gases. Normal LCI in children is around six turnovers of FRC volume but can vary slightly depending on the gas selected (Nuttall et al., 2021). Abnormally high LCI results directly from the delayed time constant in the poorly ventilated lung areas.

LCI is recognized as a more sensitive marker than the pulmonary function test (PFT) in detecting airway disease in patients with cystic fibrosis and primary ciliary dyskinesia (Kinghorn et al., 2020). LCI is also reported to be abnormal in children with severe asthma, but within the normal range in children with mild‐to‐moderate asthma (Nuttall et al., 2021; Usemann et al., 2017). Even though LCI is normal in children with stable asthma, it remains significantly higher than that of healthy controls (Zwitserloot et al., 2014).

Unlike the case in asthma and other obstructive lung diseases, very limited studies have investigated the effect of obesity on worsening ventilation inhomogeneity as a mechanism by which obesity can worsen asthma. None of these studies have measured LCI in individuals with obesity. We aimed to measure LCI in overweight/obese versus normal weight children with and without asthma in a four‐group cross‐sectional comparative design. We also aimed to evaluate the direct relationship between BMI, airway dysanapsis, airway obstruction, airway inflammation, and LCI in these children. We hypothesized that LCI is higher in children with obesity and with or without asthma compared to their disease controls due to airway dysanapsis, airway obstruction, or low resting lung volumes. We also hypothesized that obesity correlates with elevated LCI, potentially interacting positively with asthma.

## 2 MATERIALS AND METHODS

This study is an arm of the Sphingolipids in Obesity and Asthma in Pediatrics (SOAP) study, which is a large multipurpose project with a cross‐sectional design that aims to investigate physiological, genetic, epigenetic, metabolomic, and lipidomic factors affecting obesity and asthma in children in the state of Qatar. The study was approved by our institution's review board (IRB No. 1500770). The detailed protocol of the original study is explained elsewhere (Antonisamy et al., 2023). Briefly, study subjects (age 6–17 years) were recruited from general pediatric, pediatric pulmonary, and pediatric endocrinology clinics at Sidra Medicine Hospital, Doha, Qatar, between August 2017 and December 2022.

After consent was obtained, a detailed medical history was collected, and a physical examination was conducted to determine the subject's eligibility. Eligible subjects were then assigned to one of four groups based on their body weight and asthma diagnosis as follows: (1) normal weight with asthma (NW‐A) group, (2) overweight/obesity with asthma (OO‐A) group, (3) overweight/obesity with no asthma (OO) group, and (4) normal weight with no asthma (NW) group. Overweight and obesity were defined as BMI above the 85th and 95th percentiles (World Health Organization), respectively, for age and gender. The diagnosis of asthma was determined by the treating physician. Subjects were included in one of the asthma groups only if the asthma diagnosis was made at least 6 months prior to recruitment and if the subject had a history of asthma symptoms according to the Global Initiative of Asthma (GINA) guidelines, regardless of previous lung function test results (Levy et al., 2023).

Subjects were excluded if they were diagnosed with any of the following chronic lung diseases: cystic fibrosis, primary ciliary dyskinesia, congenital lung disease, chest wall deformities, or neuromuscular diseases. Subjects with congenital heart disease, immune deficiency, autoimmune diseases, cancer, or inborn errors of metabolism were also excluded. Subjects with obesity associated with Down syndrome, Prader‐Willie syndrome, or endocrinopathy were also excluded. Patients with a history of acute asthma exacerbation in the month preceding recruitment were excluded.

Within the first week of enrollment, the following tests were performed on all participating subjects: Pulmonary function testing (PFT), body plethysmography, LCI, and fractional exhaled nitric oxide (FeNO). Airway dysanapsis was defined as FVC >80% predicted, FEV1 >80% predicted, and FEV1/FVC ratio <80% predicted.

Details of testing procedures are as follows:

### 2.1 Pulmonary function testing (spirometry and body plethysmography)

Pulmonary function testing (PFT) was performed according to American Thoracic Society (ATS) guidelines using the Jaeger MS‐PFT Analyzer (CareFusion, Leibnizstrasse, Germany) (Antonisamy et al., 2023). PFT predicted values and *z*‐scores were calculated based on Global Lung Initiative (GLI) reference data for normal subjects (Cooper et al., 2017). Body plethysmography was also conducted according to the ATS guidelines using the same machine (Antonisamy et al., 2023).

### 2.2 Lung clearance index (LCI) and fractional exhaled nitric oxide (FeNO) measurements

LCI was measured using the multiple breath nitrogen (N_2_) washout (MBNW) technique using Ecomedic's Exhalyzer CLD 88 (Switzerland) and SPIROWARE v 3.2.1. The subject was asked, while seated, to start tidal breathing of 100% oxygen (O_2_) through a mouthpiece connected to a pneumotach and nitrogen (N_2_) gas analyzer with the nose closed by a clip. Breath‐by‐breath measurement of end‐tidal N_2_ was obtained from the sampling side port until N_2_ dropped to 2.5% from the baseline. Pressure pneumotach was used to calculate airflow signal, which was integrated to obtain total exhaled volumes and calculate FRC. LCI is a measure of the number of FRC turnover required to washout 97.5% of the lung's nitrogen and was calculated by dividing the cumulative exhaled volume by FRC (Jensen et al., 2013). Each subject performed at least three LCI trials. A coefficient of variance of <10% between consecutive trials was accepted. The average of at least two accepted trials was included in the analysis. LCI z‐scores were calculated based on GLI normative values (Ramsey et al., 2024). The recently published GLI *z*‐scores were based on LCI measurements obtained using a newer soft version (SPIROWARE v 3.3.1), which has different N_2_ sensitivity and calculation algorithms. Therefore, LCI data for all the study subjects were migrated to the updated software and recalculated as LCI (GLI) in order to obtain LCI (GLI) *z*‐scores. LCI and LCI (GLI) strongly correlated in the entire study population (data not shown). However, LCI (GLI) was around 10% lower than LCI. All statistical analyses were based on LCI data obtained using the older software version. Only *z*‐score analyses were based on data obtained using the updated software.

Furthermore, phase 3 slopes of single breath N_2_ washout affected by ventilation at the acinar (Sacinar) or conductive (Scond) regions were also collected during LCI measurement. *Z*‐scores for Sacinar and Scond were calculated based on Houltz and Gustafsson, 2012 (unpublished data provided by equipment manufacturer).

FeNO was measured using the same instrument (Ecomedic's Exhalyzer CLD 88, Switzerland) with the NO gas analyzer. The subject was asked to breathe through the same mouthpiece connected to a pneumotach and the NO gas analyzer for at least 6 seconds while maintaining constant predetermined airflow using a visual aid. Each subject performed at least three trials. Measurements were accepted if airflow was maintained within the predetermined limits. The average value of at least two accepted trials was included in the analysis.

### 2.3 Statistical analysis

Demographic and clinical data were summarized as means and standard deviations (SD) if normally distributed, and as medians and interquartile ranges (IQR) if not normally distributed. Continuous variables were compared between the four groups using one‐way analysis of variance (ANOVA) or the Kruskal–Wallis test. Box plots and pairwise *p*‐values were used to present multiple comparisons of the Bonferroni test. Skewed continuous variables were log‐transformed. Categorical variables were summarized as counts and percentages. The chi‐square test was used for group comparisons. The sample size was calculated based on pilot data examining the serum ceramide/dihydroceramide ratio in children. We needed 90 subjects per group to detect a difference in the mean ratios of 0.25 (unitless ratio) between nonatopic asthmatic (mean 1.47) and nonatopic and nonasthmatic (mean 1.22) children, with a standard deviation of 0.6 at 80% power and a 5% level of significance.

Scatter plots and Pearson or Spearman rank correlations were performed to correlate LCI with FeNO, FEV1% predicted, FEV1/FVC, FEF25–75% predicted, and RV/TLC for the entire population. Multiple linear regression analysis was used to evaluate potential predictors of LCI, including age, gender, asthma status (yes/no), BMI, and FeNO. Logistic regression analysis was performed to evaluate the effect of overweight/obesity versus asthma on airway dysanapsis.

Multiple imputation method was utilized to handle missing data using the multivariate imputation by chained equations (MICE) algorithm. Multiple linear regression analyses with missing and imputation data were reported for comparison and inference purposes. A *p*‐value <0.05 was considered statistically significant. All the statistical analyses were performed using STATA 18/SE (StataCorp LLC, College Station, Texas, USA) software.

## 3 RESULTS

A total of 364 subjects were recruited and assigned to the four groups as follows: 97 subjects in the normal weight with asthma (NW‐A) group, 100 subjects in the overweight/obese with asthma (OO‐A) group, 100 subjects in the overweight/obese with no asthma (OO) group, and 67 subjects in the normal weight with no asthma (NW) group. There was no significant difference in age between the NW‐A and NW groups. Similarly, there was no difference in age between the OO‐A and OO groups. However, the overweight/obese groups were older than the normal weight groups after controlling for asthma status (*p* < 0.001) (Table 1). The overweight/obese groups had higher BMI and BMI Z‐score than the normal weight groups after controlling for asthma status (*p* < 0.001) (Table 1). The asthma groups had a higher male/female ratio, history of eczema, and allergic rhinitis than the nonasthmatic groups after controlling for overweight/obesity status (*p* < 0.001).

**TABLE 1:** Basic characteristics of study participants (*n* = 364).

Variables	Normal weight and asthma (NW‐A)	Overweight/obesity and asthma (OO‐A)	Overweight/obesity (OO)	Normal weight (NW)	*p* Value^a^
	(*n* = 97)	(*n* = 100)	(*n* = 100)	(*n* = 67)	
Age (years)^b^	10.87 (3.1)	12.04 (3.4)	12.40 (2.9)	10.67 (3.1)	<0.001
Gender, *n* (%)					
Male	72 (74.2)	73 (73.0)	45 (45.0)	36 (53.7)	<0.001
Female	25 (25.8)	27 (27.0)	55 (55.0)	31 (46.3)	
BMI (kg/m^2^)^b^	17.18 (2.7)	27.20 (5.9)	32.07 (8.9)	16.77 (2.3)	<0.001
BMI (Z score)^c^	−0.14 (−0.8,0.5)	1.93 (1.5,2.3)	2.18 (1.8,2.6)	−0.31 (−0.9,0.2)	<0.001
BMI percentile for age^c^	44.5 (21.9, 69.1)	97.3 (93.6, 98.9)	98.5 (96.7, 99.5)	38.0 (16.5, 58.9)	<0.001
Eczema, *n* (%)	35 (36.1)	23 (23.0)	9 (9.0)	0 (0.0)	<0.001
Allergies, *n* (%)	35 (36.1)	31 (31.0)	7 (7.0)	1 (1.5)	<0.001
Rhinitis, *n* (%)	45 (46.4)	51 (51.0)	3 (3.0)	2 (3.0)	<0.001
Medication					
Montelukast, *n* (%)	27 (27.8)	33 (33.0)	–	–	0.43
Inhaled steroids, *n* (%)	31 (32.0)	31 (31.0)	–	–	0.88
Inhaled steroids and LABA, *n* (%)	17 (17.5)	20 (20.0)	–	–	0.66

Abbreviation: LABA, Long‐Acting Beta2‐Agonists.

^a^ *p* Value is based on comparing groups using one‐way ANOVA or the Kruskal–Wallis test.

^b^ Mean (SD) for normally distributed variables.

^c^ Median (IQR) for skewed variables.

Pulmonary function testing showed no significant differences in FVC% predicted between all the study groups. Airway obstruction parameters, including FEV1% predicted, FEV1/FVC% predicted, and FEF 25–75% predicted, were lower in the NW‐A group than in the NW group (*p* = 0.002, *p* < 0.001, *p* < 0.001, respectively). These parameters were also lower in the OO‐A group than in the OO group (*p* < 0.001, *p* < 0.001, and *p* < 0.001, respectively). However, there was no difference in the same parameters between the OO‐A and NW‐A groups. Also, there was no difference in the same parameters between the OO and NW groups (Table 2).

**TABLE 2:** Pulmonary function test parameters.

Variables	Normal weight and asthma (NW‐A)	Overweight/obesity and asthma (OO‐A)	Overweight/obesity (OO)	Normal weight (NW)	Asthma related		Obesity related	
	Group A	Group B	Group C	Group D	A vs. D	B vs. C	A vs. B	C vs. D
	(*n* = 96)	(*n* = 100)	(*n* = 97)	(*n* = 66)	*p* Value	*p* Value	*p* Value	*p* Value
Spirometry (*n* = 359)								
FVC (L)^a^	2.44 (1.0)	2.87 (1.0)	3.11 (0.8)	2.35 (0.9)	1.0	0.45	0.009	<0.001
FVC % predicted^a^	94.18 (13.2)	98.47 (13.4)	100.19 (12.5)	94.27 (12.2)	1.0	1.0	0.12	0.026
FVC z‐score^b^	−0.4 (−1.1, 0.4)	−0.1 (−0.9, 0.6)	0.1 (−0.8, 0.7)	−0.5 (−1.3, −0.1)	1.0	0.89	0.67	0.009
FEV1 (L)^a^	1.95 (0.8)	2.26 (0.8)	2.67 (0.7)	2.07 (0.8)	1.0	0.001	0.028	<0.001
FEV1% predicted^a^	86.40 (15.3)	89.53 (16.7)	98.93 (12.2)	94.74 (12.4)	0.002	<0.001	0.78	0.42
FEV1 z‐score^b^	−1.0 (−1.9, −0.2)	−0.8 (−1.8, 0.2)	−0.1 (−0.8, 0.5)	−0.5 (−1.1, 0.0)	0.012	<0.001	1.0	0.14
FEV1/FVC ratio^a^	80.15 (9.4)	78.97 (8.9)	86.15 (6.4)	88.02 (5.1)	<0.001	<0.001	1.0	0.83
FEV1/FVC ratio % predicted^a^	91.22 (10.4)	90.18 (10.0)	98.46 (6.9)	100.08 (5.9)	<0.001	<0.001	1.0	1.0
FEV1/FVC z‐score^b^	−1.0 (−2.0, 0.0)	−1.0 (−2.0, 0.0)	0.0 (−1.0, 0.0)	0.0 (0.0, 1.0)	<0.001	<0.001	1.0	0.78
FEF25–75% (L/s)^a^	1.82 (0.9)	2.11 (0.9)	2.96 (1.0)	2.36 (1.0)	0.003	<0.001	0.19	0.001
FEF 25–75% % predicted^a^	67.66 (26.9)	70.99 (25.6)	91.62 (19.9)	87.93 (21.4)	<0.001	<0.001	1.0	1.0
FEF25–75% *z*‐score^b^	−1.6 (−2.2, −0.7)	−1.2 (−2.2, −0.5)	−0.2 (−1.1, 0.3)	−0.7 (−1.4, 0.1)	<0.001	<0.001	0.65	0.41
Plethysmography (*n* = 318)								
sRaw^a^	1.10 (0.4)	1.22 (0.6)	0.97 (0.4)	0.94 (0.2)	0.12	<0.001	0.34	1.0
sRaw % predicted^b^	196.2 (160.1, 244.9)	217.0 (171.2, 266.7)	173.15 (144.5, 211.4)	177.1 (148.7, 209.8)	0.08	<0.001	0.40	1.0
FRCpleth^a^	2.03 (0.8)	1.97 (0.6)	1.97 (0.5)	1.80 (0.6)	0.24	1.0	1.0	0.66
FRCpleth % predicted^b^	111.6 (99.0, 125.7)	101.9 (90.9, 111.8)	93.45 (84.4, 109.0)	105.4 (93.7, 119.3)	0.37	0.09	0.003	0.001
FRCpleth z‐score^b^	1.1 (−0.1, 2.1)	0.2 (−0.8, 1.0)	−0.5 (−1.3, 0.6)	0.5 (−0.5, 1.6)	0.37	0.09	0.002	0.001
RV^a^	0.99 (0.3)	0.94 (0.3)	0.82 (0.3)	0.90 (0.3)	0.64	0.047	1.0	0.46
RV % predicted^b^	109.7 (91.1, 128.2)	100.55 (80.4, 119.7)	81.3 (68.8, 93.5)	107.8 (89.4, 121.1)	1.0	<0.001	0.034	<0.001
RV z‐score^b^	0.4 (−0.4, 1.2)	0.0 (−0.8, 0.8)	−0.8 (−1.3, −0.3)	0.3 (−0.4, 0.9)	1.0	<0.001	0.033	<0.001
TLC^a^	3.70 (1.2)	4.17 (2.3)	4.17 (0.9)	3.47 (1.1)	1.0	1.0	0.31	0.030
TLC % predicted^b^	96.1 (89.5, 103.4)	96.2 (88.5, 103.4)	93.9 (85.4, 102.8)	90.2 (85.5, 99.4)	0.13	0.52	1.0	1.0
TLC z‐score^b^	−0.5 (−1.3, 0.3)	−0.5 (−1.5, 0.2)	−0.7 (−1.5, 0.3)	−1.0 (−1.7, −0.1)	0.23	1.0	1.0	1.0
RV/TLC^a^	28.47 (6.4)	25.56 (8.4)	21.07 (5.9)	28.57 (5.6)	1.0	<0.001	0.042	<0.001
RV/TLC % predicted^b^	111.7 (97.0, 126.8)	100.1 (85.8, 114.0)	87.9 (70.3, 99.6)	113.0 (99.2, 129.0)	1.0	<0.001	0.003	<0.001
RV/TLC z‐score^b^	0.6 (−0.2, 1.4)	0.0 (−0.7, 0.7)	−0.6 (−1.5, 0.0)	0.6 (0.0, 1.5)	1.0	<0.001	0.003	<0.001
LCI (*n* = 308)								
LCI^a^	7.23 (1.3)	7.33 (1.8)	6.66 (0.8)	6.57 (0.6)	0.017	0.003	1.0	1.0
LCI (GLI)^a^	6.48 (1.1)	6.63 (1.5)	6.17 (0.7)	6.02 (0.5)	0.068	0.019	1.0	1.0
LCI z‐score (GLI)^b^	0.07 (−0.9, 0.9)	0.03 (−0.6, 1.1)	−0.39 (−1.2, 0.5)	−0.50 (−1.3, 0.2)	0.016	0.047	1.0	0.7
Scond^b^	0.034 (0.02, 0.05)	0.039 (0.03, 0.06)	0.028 (0.02, 0.04)	0.019 (0.01, 0.03)	<0.001	0.001	0.15	0.001
Scond z‐score^b^	3.5 (0.7, 5.6)	4.5 (2.1, 6.3)	2.4 (1.1, 3.6)	0.6 (−1.8, 2.1)	<0.001	<0.001	0.19	0.004
Sacin^b^	0.08 (0.05, 0.11)	0.049 (0.03, 0.07)	0.041 (0.03, 0.06)	0.065 (0.04, 0.08)	0.31	0.72	0.001	0.026
Sacin z‐score^b^	0.9 (−0.6, 2.1)	−0.6 (−2.2, 0.8)	−1.0 (−2.8, 0.3)	0.6 (−0.9, 1.5)	0.90	0.64	0.004	0.008
FeNO (*n* = 330)								
FeNO^b^	43.90 (19.4, 73.6)	34.35 (16.3, 80.1)	15.40 (10.6, 27.0)	17.90 (11.8, 29.3)	<0.001	<0.001	1.0	1.0

Abbreviations: FEF25–75%, forced mid‐expiratory flow; FeNO, fractional exhaled nitric oxide; FEV1, forced expiratory volume in the first second; FRCpleth, functional residual capacity measured by plethysmography; FVC, forced vital capacity; LCI(GLI), LCI measured using SPIROWARE v3.3.1 and used for z‐score calculation based on GLI 2024 normal values; LCI, Lung clearance Index measured using SPIROWARE v3.2.1; RV, residual volume; Sacin, phase III slope avererage which reflects ventilation inhomogeneity at acinar level; Scond, phase III slope avererage which reflects ventilation inhomogeneity at conductive airway level; sRaw, specific airway resistance; TLC, total lung capacity.

^a^ Mean (SD) for normally distributed variables.

^b^ Median (IQR) for skewed variables; *p* values for pairwise differences using Bonferroni multiple comparison test from one‐way ANOVA or Dunn test. Post hoc analysis values were provided to isolate the asthma effect from the obesity effect.

Resting lung volumes, including FRC% predicted, RV% predicted, RV/TLC, and RV/TLC% predicted, were lower in the OO group than in the NW group (*p* = 0.001, *p* < 0.001, *p* < 0.001, and *p* < 0.001 respectively). Resting lung volumes were also lower in the OO‐A group than in the NW‐A group (*p* = 0.003, *p* = 0.034, *p* = 0.042, and *p* = 0.003, respectively). On the other hand, RV% predicted, RV/TLC, and RV/TLC% predicted were higher in the OO‐A group compared to the OO group (*p* < 0.001, *p* < 0.001, and *p* < 0.001, respectively), but there was no significant difference in the same parameters between the NW‐A and NW groups (Table 2).

**FIGURE 1:**
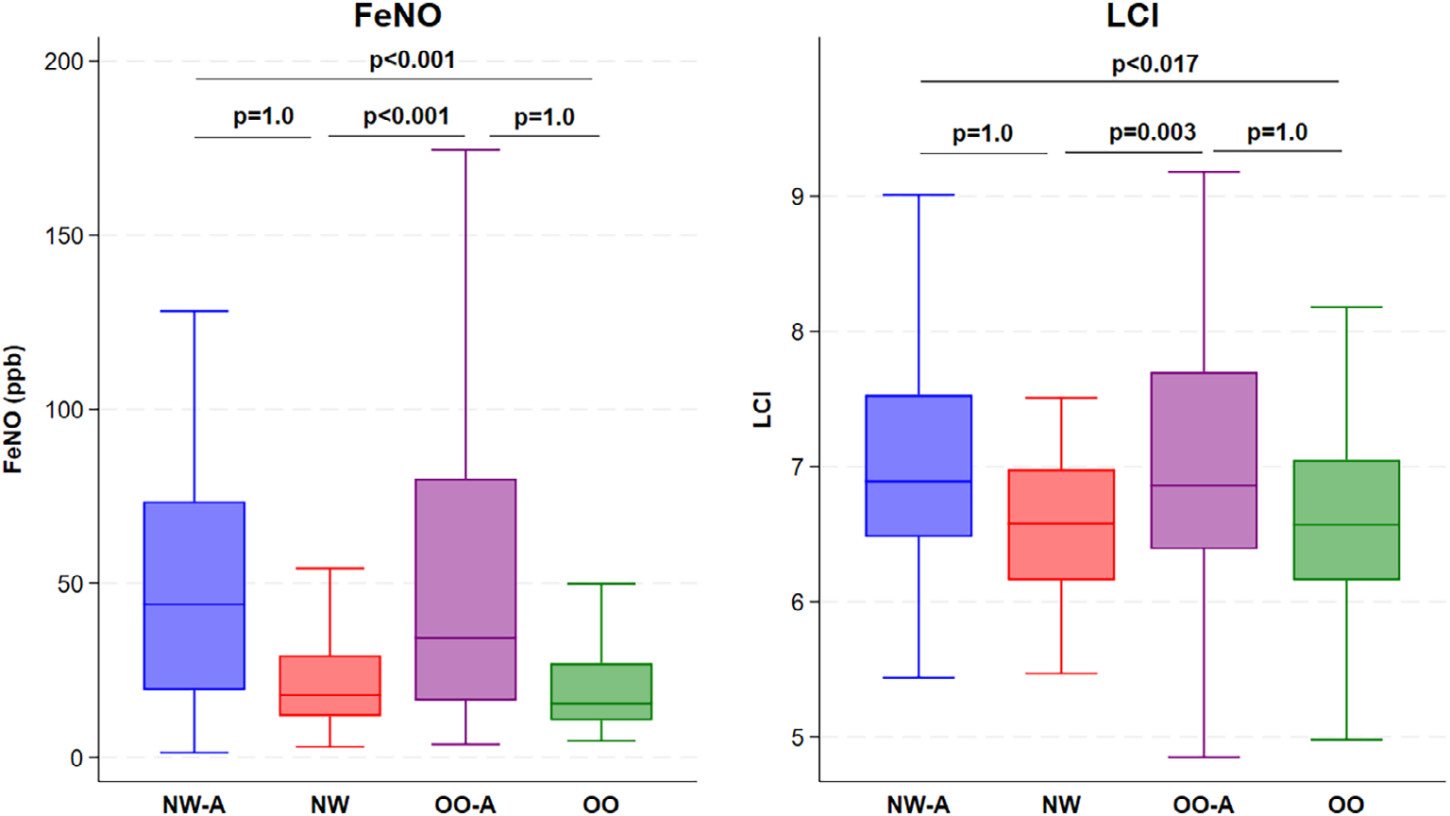
Box plot for differences in FeNO and LCI between the four study groups (NW, normal weight with no asthma; NW‐A, normal weight with asthma; OO, overweight/obese with no asthma; and OO‐A, overweight/obese with asthma).

LCI was not significantly different between the OO and NW groups. There was also no difference in LCI between the OO‐A and NW‐A groups (Table 2) (Figure 1). However, LCI was higher in the NW‐A group than in the NW group (*p* = 0.017). Similarly, LCI was higher in the OO‐A group than in the OO group (*p* = 0.003). Scond was significantly higher in the NW‐A group compared to the NW group (*p* < 0.001) and in the OO‐A group compared to the OO group (*p* < 0.001). There was no difference in Scond between the OO‐A and NW‐A groups. However, Scond was significantly higher in the OO group compared to the NW group (*p* = 0.001).

Sacinar was significantly lower in the OO‐A group compared to the NW‐A group (*p* = 0.001), and was significantly lower in the OO group compared to the NW group (*p* = 0.026). There was no significant difference in Sacinar between the OO‐A group and the OO group, and no difference between the NW‐A group and the NW group.

**FIGURE 2:**
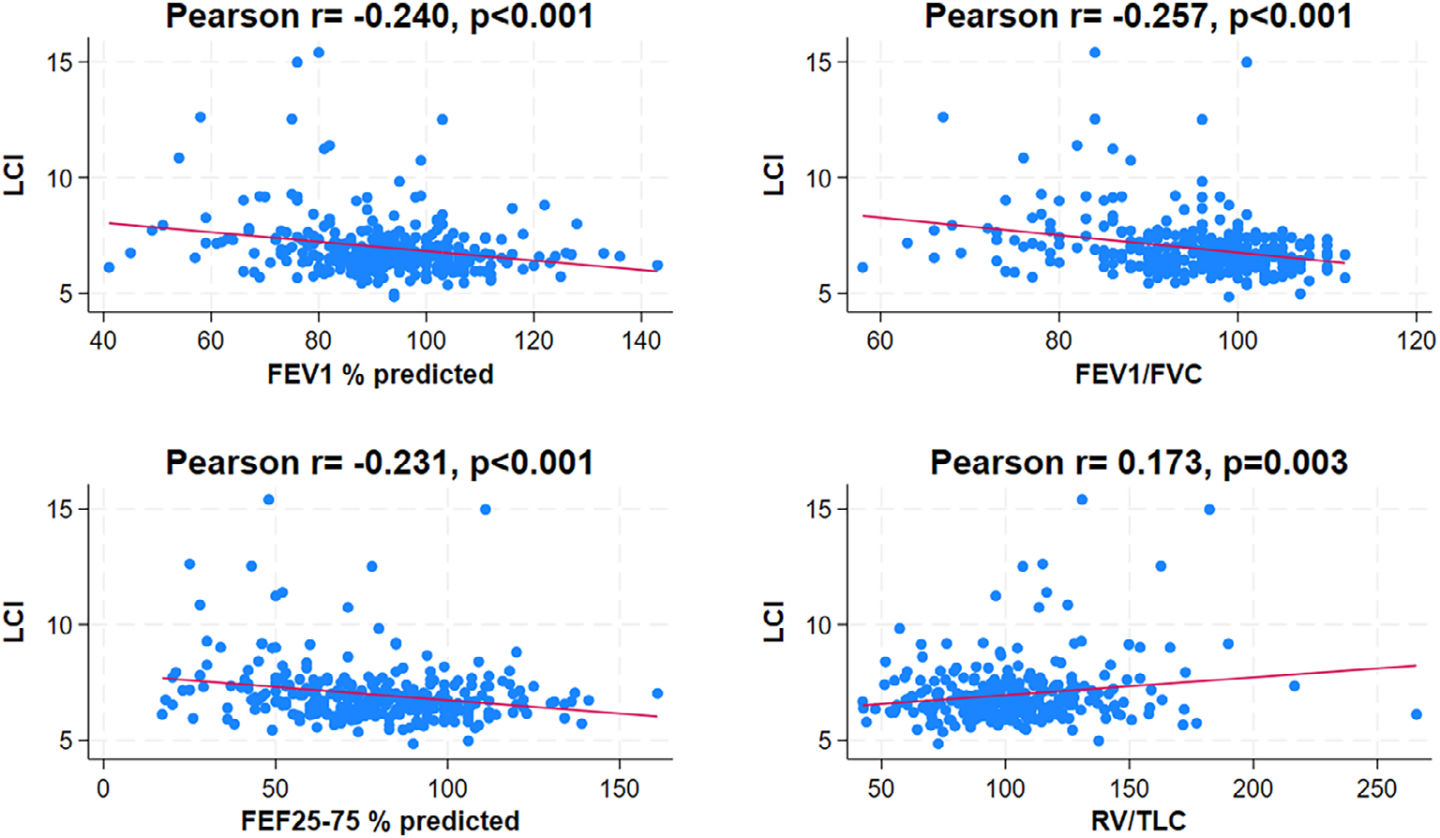
Scatterplot and correlations between LCI and the PFT parameters for the entire study population (FEV1% predicted, FEV1/FVC% predicted, FEF25–75% predicted, and RV/TLC).

**FIGURE 3:**
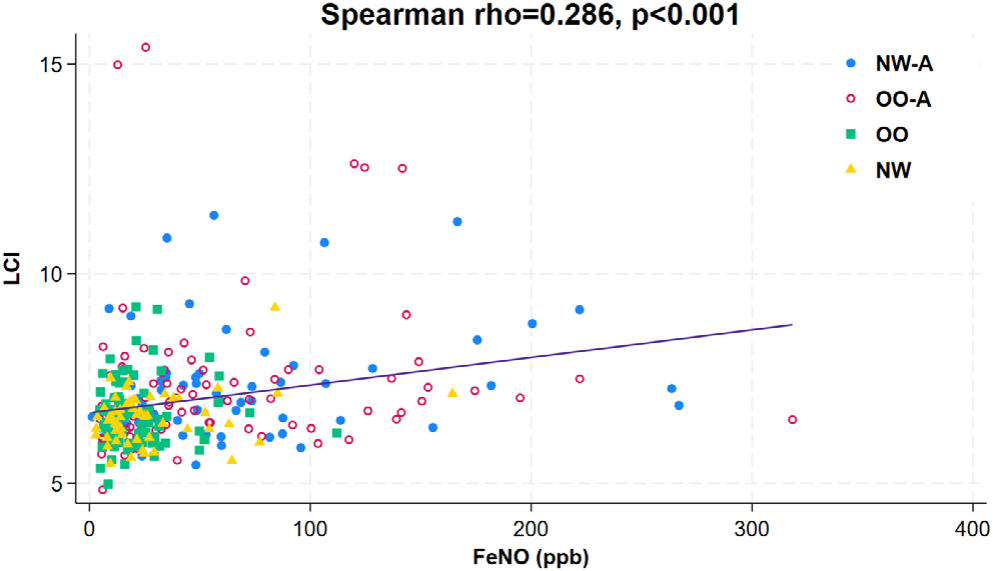
Scatterplot and correlation between LCI and FeNO for the entire study population (NW, normal weight with no asthma; NW‐A, normal weight with asthma; OO, overweight/obese with no asthma; and OO‐A, overweight/obese with asthma).

We found no correlation between LCI and BMI in the entire population (Table 3). However, there was a significant negative correlation between LCI and airway obstruction parameters, including FEV1% predicted (*r* = −0.24, *p* < 0.001), FEV1/FVC% predicted (*r* = −0.26, *p* < 0.001), and FEF25–75% predicted (*r* = −0.23, *p* < 0.001) (Figure 2). Conversely, LCI negatively correlated with RV/TLC (*r* = 0.17, *p* = 0.003) (Figure 2). There was also a positive correlation between LCI and FeNO (*r* = 0.29, *p* < 0.001) (Figure 3).

**TABLE 3:** Multiple regression analysis for Lung Clearance Index (LCI)as the dependent variable with age, gender, asthma, BMI, and FeNO as the independent variables.

Variables	Complete cases (*n* = 293)		Multiple imputation (*n* = 364)	
	*β* (95% CI)	*p* Value^a^	*β* (95% CI)	*p* Value^a^
Age, years	−0.0332 (−0.09, 0.02)	0.22	−0.0283 (−0.08, 0.03)	0.29
Gender (male)	0.0236 (−0.28, 0.33)	0.88	0.0272 (−0.29, 0.34)	0.86
Asthma (yes)	0.5128 (0.19, 0.84)	0.002	0.4953 (0.19, 0.80)	0.002
BMI (kg/m^2^)	0.0179 (−0.001, 0.04)	0.06	0.0173 (−0.001, 0.04)	0.06
FeNO (ppb) – log transformed	0.2861 (0.12, 0.46)	0.001	0.2749 (0.09, 0.46)	0.004

*Note*: Estimates of effect size are presented as regression coefficients (*β*) and 95% confidence interval. β‐values indicate the change in the outcome per unit change in the predictor variable.

Abbreviations: BMI, body mass index; FeNO, fractional exhaled nitric oxide.

^a^ Models were adjusted for age, gender, asthma, BMI, and FeNO for the complete cases and imputed data.

**FIGURE 4:**
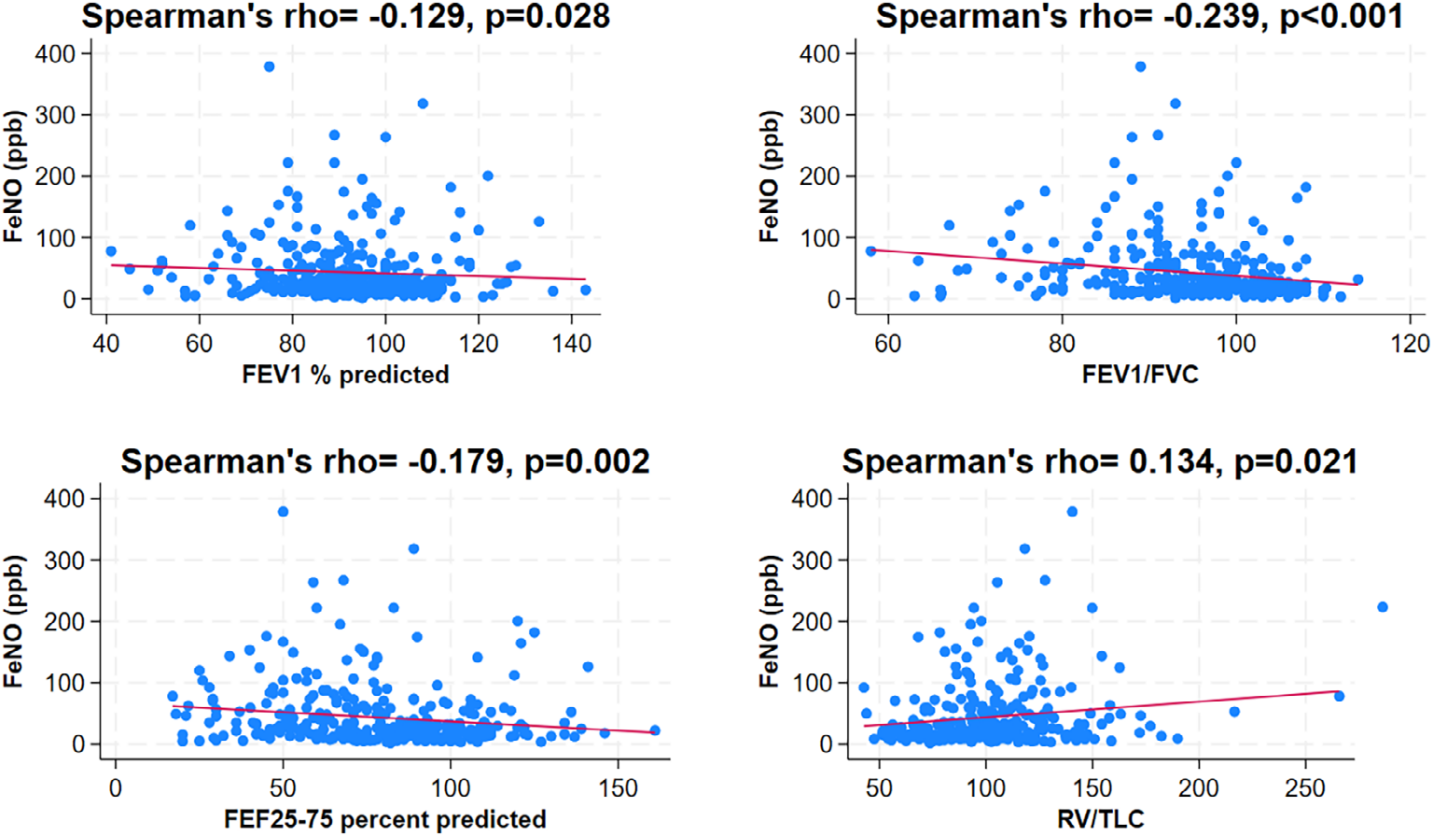
Scatterplot and correlation between FeNO and the PFT parameters (FEV1% predicted, FEV1/FVC% predicted, FEF 25–75% predicted, and RV/TLC) for the entire study population (NW, normal weight with no asthma; NW‐A, normal weight with asthma; OO, overweight/obese with no asthma; and OO‐A, overweight/obese with asthma).

FeNO was higher in the OO‐A group than in the OO group (*p* < 0.001). Also, FeNO was higher in the NW‐A group as compared to the NW group (*p* < 0.001) (Table 2) (Figure 1). There was a significant negative correlation between FeNO and parameters of airway obstruction, including FEV1% predicted (*r* = −0.13, *p* = 0.028), FEV1/FVC% predicted (*r* = −0.24, *p* < 0.001), and FEF25–75% predicted (*r* = −0.18, *p* = 0.002). Also, there was a significant positive correlation between FeNO level and RV/TLC ratio (*r* = 0.13, *p* = 0.021) (Figure 4).

Multivariable regression analysis showed that asthma diagnosis (*p* = 0.002) and high FeNO level (*p* = 0.004) were the only significant predictors of high LCI in the entire population. BMI was not a significant predictor of LCI (*p* = 0.06) (Table 3). The interaction between asthma diagnosis and BMI was not significant. Logistic regression analysis showed that asthma was the only significant risk factor for airway dysanapsis. Obesity, on the other hand, was not a significant predictor of dysanapsis (Table 4).

**TABLE 4:** Logistic regression analysis evaluating predictor of dysnapsis in the study population.

Dysnapsis	OR (95% CI)	*p* Value
Asthma	5.8 (2.6, 12.9)	<0.001
Obesity	1.3 (0.7, 2.4)	0.440

*Note*: Model was adjusted for age and gender.

## 4 DISCUSSION

In this study, we aimed to evaluate the effect of obesity on lung ventilation inhomogeneity and its potential role in worsening airway dysfunction in children with and without asthma. The study was uniquely designed to evaluate the effect of obesity and asthma separately and in combination on lung ventilation inhomogeneity by utilizing a cross‐sectional four‐group design and comparing the lung clearance index (LCI) between these groups.

Contrary to our hypothesis, obesity was not associated with increased inhomogeneity of lung ventilation regardless of asthma status. LCI was not different between the children with overweight/obesity and normal weight children with no asthma. Also, LCI was not different between children with overweight/obesity and asthma, and those with normal weight and asthma. Furthermore, BMI was not a significant predictor of high LCI in the entire population, and there was no interaction between asthma and obesity (BMI) in predicting high LCI. Therefore, obesity is unlikely to affect asthma pathophysiology by causing or worsening the inhomogeneity of lung ventilation.

In order to evaluate if ventilation inhomogeneity is affected by obesity at the conductive airway level (i.e., convective air motion) versus acinar level (i.e., diffusive airwawa motion), we measured phase 3 slope indices, namely Scond for the former and Sacinar for the latter. Interestingly, we found that overweight/obesity groups have lower Sacinar than nonobese groups. This suggests that overweight/obesity is associated with improved ventilation at the diffusive level. As expected, asthma groups had higher Scond than nonasthma groups, confirming that the high LCI seen in the asthma groups is due to ventilation inhomogeneity at the conductive airway level.

Interestingly, we found that Scond was higher in the overweight/obese group compared to the normal weight group, which suggests that obesity is associated with ventilation inhomogeneity at the conducting airways level (i.e., convective gas motion). However, this effect did not influence the overall lung clearance index. Furthermore, overweight/obese individuals with asthma did not have high Scond compared to the normal weight with asthma group. Considering that slope indices have significant reliability issues, especially in children, no certain conclusion can be derived from these results (Riley et al., 2024). Hence, further research is needed.

Asthma diagnosis, on the other hand, is a significant predictor of high LCI. We found that high LCI was associated with asthma diagnosis, airway obstruction parameters, air trapping, and airway inflammation. Previous studies have reported high LCI in children with asthma, especially in children with uncontrolled asthma compared to controlled asthma (Keen et al., 2011; Kinghorn et al., 2020; Usemann et al., 2017). Macleod et al. showed that even children with controlled asthma have higher LCI compared to children without asthma (6.69 vs. 6.240, *p* = 0.02) (Macleod et al., 2009). Our study showed that children with asthma have higher LCI (mean LCI of 7.23 in NW‐A, and 7.33 in OO‐A) than previously reported (Keen et al., 2011; Kinghorn et al., 2020; Macleod et al., 2009). This could be attributed to the high proportion of uncontrolled asthma in our cohort, as suggested by the low percentage of asthma patients who were receiving inhaled steroids (Table 1).

Recent research showed that obesity in children is associated with “airway dysanapsis” a term used to describe relatively small airway caliber compared to normal/increased lung volume in children with obesity, which is attributed to disproportionate lung growth with respect to airway size (Arismendi et al., 2020; Forno et al., 2017). This airway dysanapsis, defined as normal FVC, normal FEV1 but low FEV1/FVC, was suggested as a possible explanation for the increased risk of asthma diagnosis and asthma morbidity in obese children (Arismendi et al., 2020; Forno et al., 2017; Jung et al., 2022). Forno et al. showed that obesity in children was associated with airway dysanapsis (OR, 1.95; 95% CI, 1.62–2.35) and correlated with high residual volume (Forno et al., 2017). Airway dysanapsis, however, was not observed in our patients with overweight/obesity. FVC, FEV1, and FEV1/FVC were not different between children with overweight/obesity and those with normal weight after controlling for asthma status. Multiple regression analysis of our entire study population showed that asthma, not overweight/obesity, as the only significant predictor of airway dysanapsis as defined above. RV and RV/TLC were lower in children with overweight/obesity compared to normal weight children after controlling for asthma status. Our findings show that obesity is associated with proportionate airway caliber but decreased residual volume. The discrepancy between our study and previous studies that confirm the presence of “airway dysanapsis” and its association with high residual volume is more likely related to the study design and patient selection. Our study suggests that “airway dysanapsis” is not likely to be found in children with overweight/obesity if asthma status is adjusted.

Unlike the case in children, inhomogeneity in lung ventilation described in adults with obesity, using measures other than LCI, was shown to be correlated with low resting lung volumes, which cause small airway narrowing or even complete closure (Pellegrino et al., 2014). Pellegrino et al. used changes in frequency‐dependent respiratory resistance using the forced oscillation technique as an indirect measure of ventilation inhomogeneity. They found that respiratory resistance was increased in obese adults only when FRC falls approximately below 65% of predicted or ERV below 0.6 L (Pellegrino et al., 2014). In our pediatric population, low resting lung volumes were also present in subjects with obesity but were not associated with decreased airway caliber as measured by FEV1, FEV1/FVC, and FEF 25–75%. A different study using a 133 xenon (Xe) ventilation scan, a crude measure of lung ventilation, found that ventilation in the upper zones of the lung was more prominent in adults with obesity who have a significant reduction in ERV (21.9% of predicted) but was uniformly distributed in those with a modest reduction in ERV (50.8% of predicted) (Holley et al., 1967). In our study, the observed reduction in resting lung volumes (FRC, RV, and RV/TLC) in children with overweight/obesity did not affect the inhomogeneity of lung ventilation as measured by LCI. Therefore, decreased lung volumes in children with overweight/obesity are unlikely to cause a significant reduction in small airway caliber or complete airway closure, leading to ventilation inhomogeneity, as in adults. Different mechanisms must be affecting asthma in children with obesity as compared to adults.

Asthma, on the other hand, has a marked impact on lung ventilation inhomogeneity as measured by LCI. Ventilation inhomogeneity in patients with asthma is directly affected by airway obstruction. Our study shows that children with asthma have a higher LCI than those without asthma, regardless of their weight status. We also found a significant correlation between airway obstruction and LCI. There was also a positive correlation between FeNO, a marker of airway inflammation, and LCI. Furthermore, asthma diagnosis and high FeNO were the only predictors of high LCI in our cohort. This association between airway obstruction, airway inflammation, and inhomogeneity of lung ventilation is supported by several studies. Keen et al. found a significant correlation between ventilation distribution measured by the slope of phase III during multiple breath washout and FeNO as well as airway hyperresponsiveness in children with asthma (Keen et al., 2011). Also, Nuttall et al. reported an inverse association between LCI and FEV1 in children with severe asthma (Nuttall et al., 2021). In contrast, Zwitserloot et al. found no significant correlation between LCI and FEF25–75% or FEV1/FVC in children with clinically stable asthma; however, inhomogeneity in lung ventilation was present in patients with asthma compared to nonasthmatics despite their normal spirometry (Zwitserloot et al., 2014).

We found a significant positive correlation between LCI and air trapping (i.e., RV% predicted and RV/TLC), which is expected since air trapping directly affects the homogeneity of lung ventilation in patients with asthma. Similar studies done in patients with cystic fibrosis, 12 years old and above, have also shown that LCI is proportionately increased with increasing trapped gas volume (Kraemer et al., 2005). Moreover, we found a significant positive correlation between FeNO and RV/TLC, as well as negative correlations between high FeNO and PFT parameters of airway obstruction. Our findings support the hypothesis that eosinophilic airway inflammation is associated with airway dysfunction, air trapping, and ventilation inhomogeneity in children with asthma. In adults with asthma, a similar strong association was also reported between FeNO and ventilation inhomogeneity, measured using single‐breath nitrogen washout test (phase III slope) (Battaglia et al., 2005). However, we found no correlation between BMI, RV/TLC, airway obstruction parameters, or FeNO, which strongly suggests that obesity in children does not cause air trapping, airway obstruction, or airway inflammation.

Our study was uniquely designed to isolate the impact of obesity versus asthma on lung clearance, airway dysfunction, lung volumes, and airway inflammation by using separate control groups based on asthma and obesity status. However, our study was limited by the relatively low number of children with morbid obesity, which prevents us from generalizing the study findings for morbid obesity. Also, we did not attempt to control for asthma severity or asthma treatments, which can be confounding. Patient selection bias can potentially confound study results. However, patient assignment to different asthma and obesity groups was based on BMI, asthma diagnosis, and asthma symptoms before any PFT, FeNO, or LCI data were obtained. In conclusion, obesity in children affects resting lung volumes but does not affect airway inflammation, airway caliber, or homogeneity of lung ventilation. Other mechanisms should be explored for the strong association between asthma and obesity in children.

## FUNDING INFORMATION

Qatar National Research Fund, Grant number: NPRP‐X‐090‐3‐037 (to Ibrahim Janahi).

## CONFLICT OF INTEREST STATEMENT

All authors declare no conflict of interest.

## INFORMED CONSENT STATEMENT

Children's assent and parents' permission form was obtained. Written informed consent has been obtained from the children and parent(s) to publish this manuscript.

## ETHICS STATEMENT

The study has been approved by the Institutional Review Board of Sidra Medicine, Doha, Qatar (7 October 2020; IRB No. 1500770).

## Data Availability

The data of this study are available from the corresponding author upon reasonable request.
